# Effectiveness of the modified Valsalva maneuver vs the standard Valsalva maneuver for the conversion of supraventricular tachycardia to normal sinus rhythm in the emergency department: a systematic review and meta analysis

**DOI:** 10.1007/s11739-026-04505-9

**Published:** 2026-08-21

**Authors:** Jared Tesch, Caleb Ruziska, Chase Urie, Ryan Brown

**Affiliations:** 1 Department of Emergency Medicine, Sanford Health, Fargo, ND USA; 2 University of North Dakota School of Medicine and Health Sciences, Grand Forks, ND USA; 3 Department of Biological Sciences, North Dakota State University, Fargo, ND USA

**Keywords:** Supraventricular tachycardia, Vagal maneuver, Modified Valsalva maneuver, Emergency meta-analysis, Standard Valsalva maneuver

## Abstract

Supraventricular tachycardia (SVT) is a cardiac arrhythmia that is commonly encountered in the emergency department (ED), for which vagal maneuvers represent first-line treatment in hemodynamically stable patients. The modified Valsalva maneuver (MVM), which involves supine repositionings and leg raises that follow the strain phase, have been proposed as a more effective alternative to the standard Valsalva maneuver (SVM). We performed a systematic review and meta-analysis (SRMA) of randomized controlled trials comparing the MVM to the SVM for conversion of SVT to normal sinus rhythm in hemodynamically stable patients presenting to the ED. PubMed, Web of Science, Google Scholar, and the Cochrane Central Register of Controlled Trials were searched from inception through March 17, 2026. Risk of bias was assessed using the Cochrane Risk of Bias 2 tool, and dichotomous outcomes were pooled as risk ratios (RR) with 95% confidence intervals (CIs). Nine trials comprising 1415 patients were included. The MVM achieved successful cardioversion in 50.1% of patients compared with 18.8% in the SVM group (polled RR 2.63, 95% CI 2.22–3.11; *I*^2^ = 0%). No major adverse events were reported in either group. The MVM is significantly more effective than the SVM for terminating SVT in the ED with no major adverse events reported in either group. These findings support the MVM as a safe, low-cost, and reproducible first-line intervention for SVT cardioversion in the ED. Further randomized trials, particularly in pediatric populations and direct comparison with pharmacologic alternatives, are warranted to confirm these results.

## Introduction

Supraventricular tachycardia (SVT) is a cardiac arrhythmia that is characterized by a rapid heart rate that originates in the upper chambers of the heart and accounts for approximately 50,000 emergency department visits each year [1]. In hemodynamically stable patients, vagal maneuvers are first-line treatment for SVT [2]. When vagal maneuvers are unsuccessful, IV adenosine or calcium channel blockers are usually next-line treatment, and show much greater efficacy, though significantly higher rates of adverse effects [3–6].

Vagal maneuvers—including Valsalva maneuvers, carotid sinus massage, bearing down, and immersion of the face in ice water—increase vagal tone and block the atrioventricular node, reducing the frequency of electrical impulses from the atria to the ventricles. Valsalva maneuvers increase intrathoracic pressure by forcefully exhaling against a closed glottis, typically by blowing into a small syringe. The modified Valsalva maneuver (MVM) increases venous return, and therefore vagal tone, by passively raising the legs of the patient to 45 degrees, and this modified technique has shown to be more effective than the standard Valsalva maneuver (SVM) [7–9].

Although prior systematic reviews and meta-analyses have demonstrated the superiority of the MVM over the SVM, they have been limited by small numbers of included trials, outdated risk-of-bias assessments, and exclusion of pediatric populations [10, 11]. To provide an updated summary and analysis of the effectiveness of the modified Valsalva compared with the standard Valsalva maneuver, we performed a systematic review and meta-analysis (SRMA).

## Methods

Patient consent and ethics approval was not applicable for this systematic review and meta-analysis as it was based on published articles. The meta analysis and systematic review was carried out following the Preferred Reporting Items for Systematic Reviews and Meta-Analyses (PRISMA) statement [12]. The aim was to evaluate the effectiveness of the modified Valsalva maneuver compared with the standard Valsalva Maneuver for the conversion of supraventricular tachycardia (SVT) to normal sinus rhythm. This review protocol was not prospectively registered.

### Search strategy and eligibility criteria

The electronic databases of Pubmed, Web of Science, Google Scholar, and Cochrane Central Register of Controlled trials were searched for studies that were published from the date of database interception and March 17, 2026 without the limitation of language. The search strategy used a combination of Medical Subject Headings (MeSH) and free-text terms for each database that include: “tachycardia, supraventricular,” “SVT,” “PSVT,” “supraventricular tachycardia,” “Valsalva maneuver," “modified Valsalva maneuver," and “modified Valsalva manoeuvre.” We also searched the bibliographic references of the included RCTs for relevant studies that may have been missed during the original search.

### Eligibility criteria

Studies were included in the systematic review and meta-analysis if they fulfilled the PICOS criteria provided (Table 1).

**Table 1:** Inclusion and exclusion criteria

Criterion	Included	Excluded
Population (P)	Hemodynamically stable adolescents and adults who presented to the emergency department (ED) with ECG confirmed supraventricular tachycardia (SVT)	Hemodynamically unstable patients. Pediatric population without adolescents. Non-SVT arrhythmias
Intervention (I)	Modified Valsalva maneuver (standardized strain followed by immediate supine repositioning and passive leg raise)	Studies not utilizing a modified Valsalva maneuver or using non-standardized or unclear techniques
Comparison (C)	Standard Valsalva maneuver (straining without postural modification)	Other vagal maneuvers (e.g., carotid sinus massage, diving reflex), pharmacologic therapies alone, or no comparator group
Outcomes (O)	At least one of the following: Conversion to sinus rhythm (primary outcome), number of attempts required, need for rescue medication, or adverse events/complications	-
Study design (S)	Randomized controlled trials (RCTs)	Observational studies, case reports, case series, conference abstracts, systematic reviews, narrative reviews, editorials, commentaries, or meta-analyses

### Study selection, data extraction, and quality assessment

Two review authors screened through the included studies and extracted data that was required for this systematic review and meta analysis in a standardized Google Sheets document. The following variables were collected: first author, year of publication, study design, country of origin, sample size, and patient demographics (including age and sex distribution where available), as well as clinical setting (e.g., emergency department or hospital).

Intervention details were extracted, including specifics of the modified Valsalva maneuver (e.g., strain pressure, duration, and postural modification such as supine repositioning and passive leg raise) and comparator characteristics (standard Valsalva maneuver technique). Additional study characteristics, including follow-up duration and number of intervention attempts, were also recorded. Disagreements between extracted data were resolved through discussion between the two reviewers.

The primary outcome was successful conversion from supraventricular tachycardia to normal sinus rhythm following the initial maneuver. Secondary outcomes include need for rescue medications and incidence of adverse events or complications.

The systematic quality of the studies was evaluated using the Cochrane Risk of Bias (RoB 2) tool [13]. Two independent reviewers assessed bias across 5 domains: bias arising from the randomization process, bias due to deviations from intended interventions, bias due to missing outcome data, bias in measurement of the outcome, and bias in selection of the reported result. Each study was classified as either low risk, some concern, or high risk of bias. Risk of bias assessments were visualized using the robvis tool [14]. Blinding was limited due to the nature of the intervention, however, outcome was considered objective due to confirmation to sinus rhythm from ECG confirmation. Disagreements were resolved through discussion between the two reviewers.

### Strategy for data synthesis

Statistical analysis was conducted using the Review Manager (RevMan) version 5.4.1. Dichotomous outcomes including successful conversion to normal sinus rhythm was pooled using risk ratio (RR) with a corresponding 95% confidence interval and displayed using a forest plot. Interstudy heterogeneity was assessed using the *I*^2^ statistic values of 0–25%, 26–75%, and >75% representing low, moderate, and high heterogeneity, respectively [15, 16]. A fixed-effect model was used for low heterogeneity, while a random-effects model was applied when moderate or high heterogeneity was present. Where applicable, additional analyses were performed for secondary outcomes, including number of attempts and adverse events. A *p*-value of <0.05 was considered statistically significant.

## Results

### Search result and general characteristics

By searching databases of PubMed, Cochrane, Google Scholar and Web of Science we uncovered 90 articles. Duplicates were screened out using Zotero. Titles and abstracts were then screened by predefined exclusion criteria noted in the PRISMA flow diagram below.

**Fig. 1:**
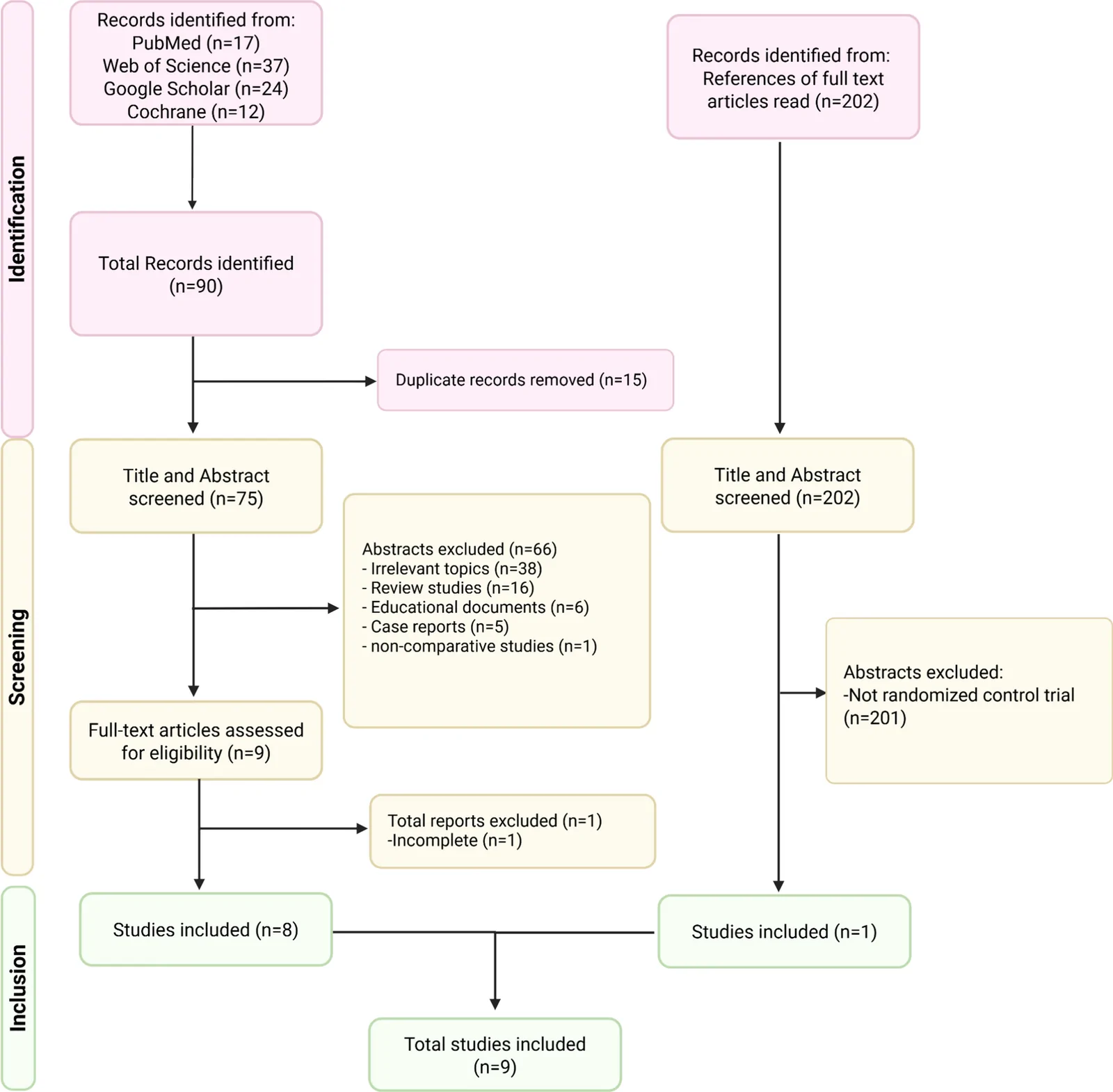
PRISMA flow diagram of the study selection process

Nine full text-articles were reviewed, and the references (if applicable) were reviewed for selection in this study. Eight of the nine full text articles were included, one ongoing study was excluded. 202 further studies were identified out of the references of these articles, and were screened similarly resulting in one additional study. This yielded a total of nine articles included in this study [17–25]. The selection process is illustrated in the PRISMA flowchart (Fig. 1), and individual study characteristics are summarized in Table 2.

**Table 2:** Characteristics and primary outcomes of included randomized controlled trials

Study	Year and country	Population	Design	*n*= MVM	*n*= SVM	MVM success (n/N)	SVM success (n/N)	% MVM	% SVM
Appelboam [17]	2015 England	Adult(18 +) Presenting to ED determined to have SVT	Total *N* = 428 Multicenter randomized controlled parallel-group trial	214	214	93/214	37/214	~43%	~17%
Çorbacıoğlu [18]	2017 Turkey	Adults 18–65 ED with PSVT	Total *N* = 56 single-center, prospective, randomized controlled trial	28	28	12/28	3/28	~43%	~11%
Ceylan [19]	2019 Turkey	Adults(18 +) who present to ED with SVT who are stable	Total *N* =98 Adults(18 +) who present to ED with SVT who are stable	32	33	14/32	8/33	~44%	~24%
Youssef [20]	2019 Egypt	Unknown ages Present to ED with SVT	Total *N* =60 Randomized control trial	30	30	14/30	6/30	~47%	~20%
Wang [21]	2020 China	Adults(18 +) with EKG confirmed PSVT	Total *N* = 361 Randomized control trial using opaque sealed envelopes	180	181	112/180	36/181	~62%	~20%
Chen [22]	2020 China	Adults 18–70 ED with SVT	*N* = 238 randomized, multicenter parallel group trial	119	119	55/119	19/119	~46%	~16%
Günaydın [23]	2021 Turkey	Adults 18+ ED with SVT	Total *N* = 47 a randomized, prospective, case–control study, single-centered	24	23	9/24	4/23	~38%	~17%
Ashraf [24]	2023 Pakistan	Adults 18–65 Hemodynamically stable ED w/SVT	Total *N* = 100 randomized control trial study	50	50	29/50	10/50	~58%	~20%
Magor [25]	2025 Egypt	Children (7–17) hemodynamically stable in ED with PSVT	Total *N*=60 Randomized controlled trial three arm	30	30	16/30	10/30	~53%	~33%

Of note, one of these articles is an abstract only and repeated searches for a full version of the article were unsuccessful [20].

### Risk of bias assessment

**Fig. 2:**
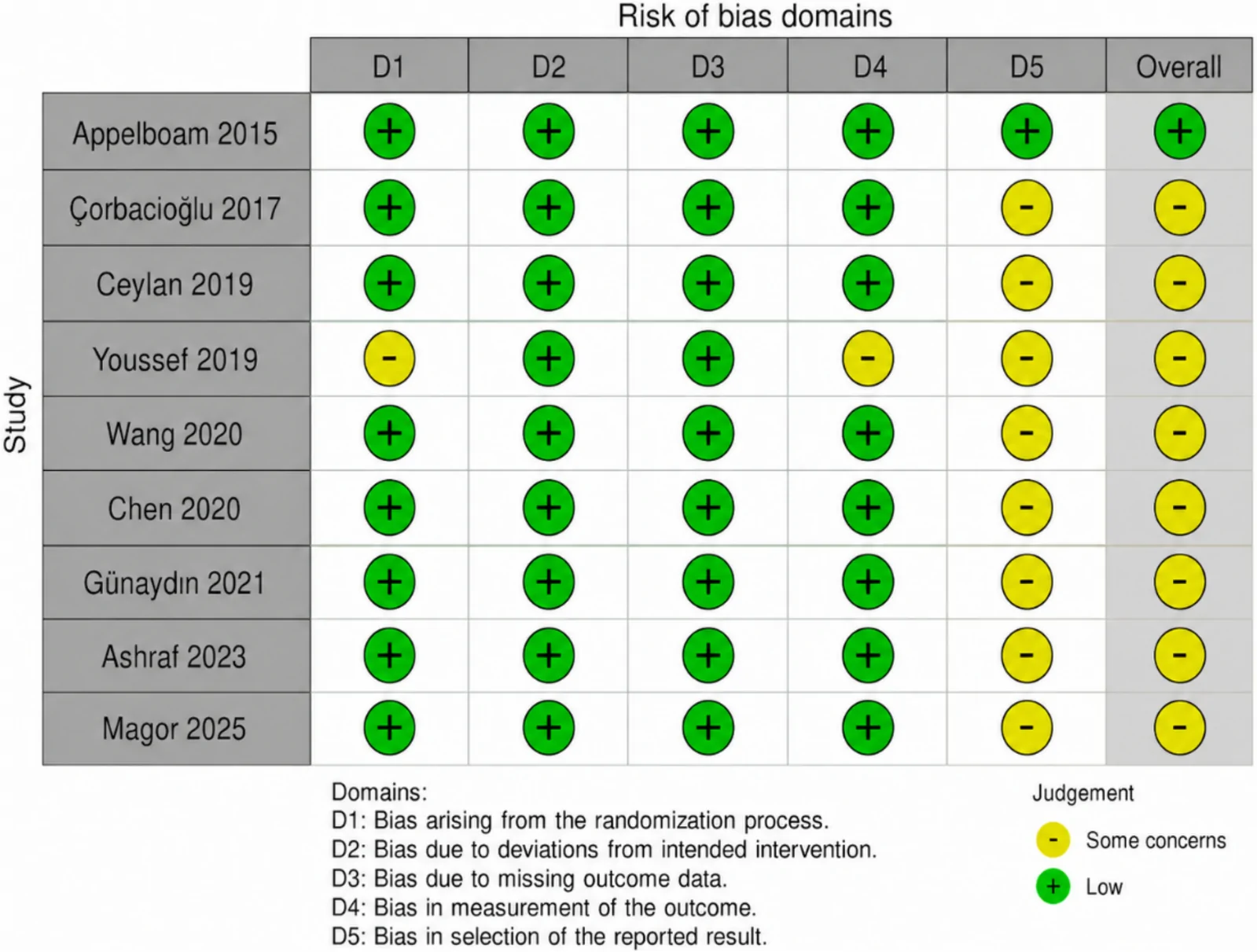
Risk of bias assessment of included randomized controlled trials using the Cochrane Risk of Bias 2 (RoB 2) tool

All nine included studies were assessed for risk of bias, and all showed low-risk or some concerns of bias influencing the results of these studies. The study with only the abstract available proved the most susceptible to risk of bias due to the sparse nature of the text [20]. Despite this, there was only some concern for this article when using the RoB 2 tool. The risk of bias assessment can be found in Fig. 2.

One study was classified as low risk because of an accessible trial protocol, demonstrating clear commitment to protocol, randomization procedures, proof of blinding whenever feasible, and providing a statistical analysis plan [17].

Minor variances in techniques used in the MVM category were noted. All studies used passive leg-raising of a supine patient as the modification, although one trial used a 90-degree leg angle rather than the 45 degrees utilized by the others [22]. In addition, one study added that one limitation of their study was a language barrier that led to “inability of the patient to understand and follow commands for performing maneuver correctly” [24]. There was also minimal variation in using syringes of varying sizes and use of a sphygmomanometer to attain ideal intrathoracic pressure.

### Primary outcome

Regarding the primary outcome—conversion to normal sinus rhythm—all studies demonstrated a higher rate of success with the MVM compared to the SVM. Seven of the nine studies showed a statistically significant difference between these figures [17, 18, 20–22, 24, 25]. Two studies were exceptions, which did still demonstrate a non-significant improvement from the SVM [19, 23]. Across all studies, a total of 354/707 patients who were selected for treatment with the MVM were successfully converted with this maneuver yielding a total success rate of 50.1% while only 133 of the 708 patients who were selected for treatment using the SVM successfully converted with this method, or about 18.8%.

**Fig. 3:**
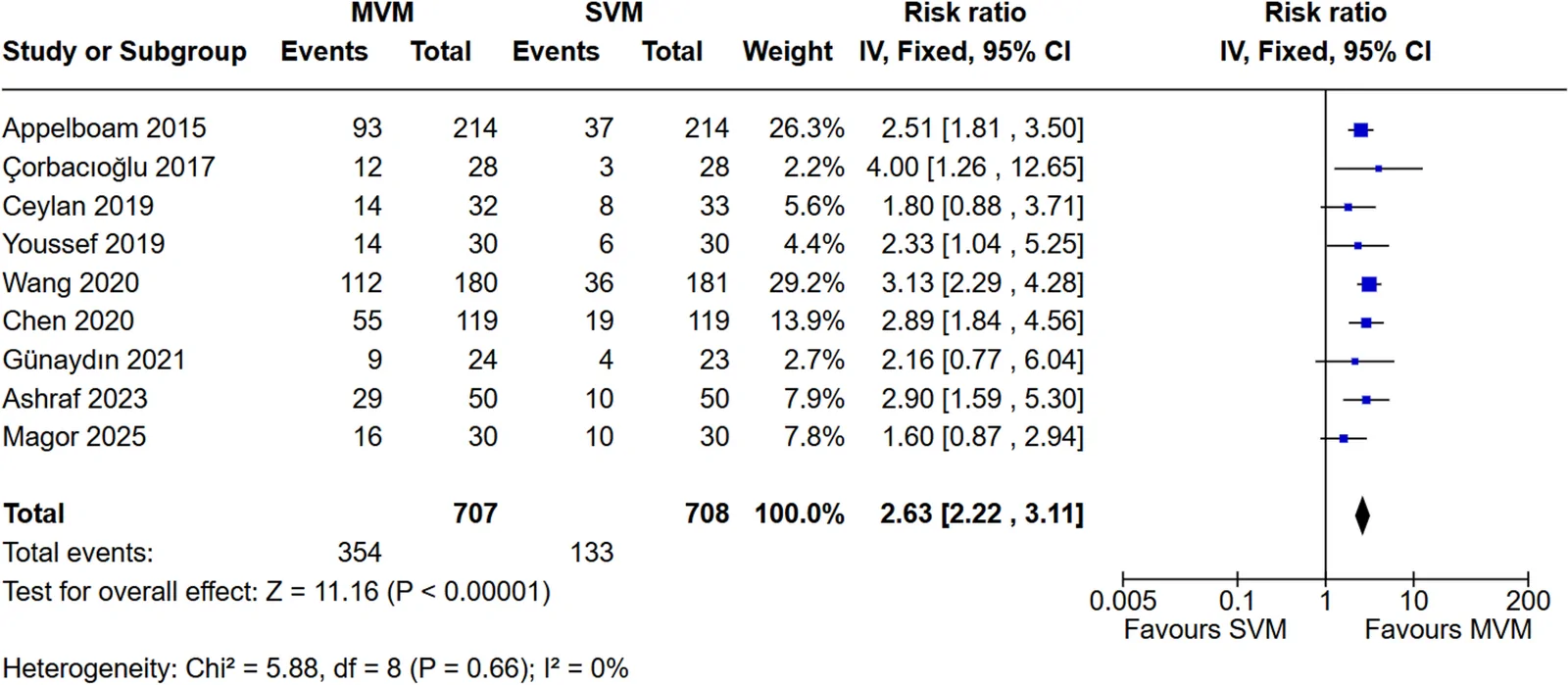
Forest plot comparing the modified Valsalva maneuver and standard Valsalva maneuver for conversion of supraventricular tachycardia to normal sinus rhythm

Meta-analysis of the data yielded a pooled risk ratio of 2.63 (95% CI 2.22–3.11), indicating that the MVM increased likelihood of cardioversion by over a factor of two compared to the SVM (Fig. 3). An *I*^2^ of 0% shows no heterogeneity, suggesting that these studies give a consistent conclusion.

No major adverse events were reported in any of the included studies. Minor adverse effects—such as nausea, dizziness, and dyspnea—were observed in both groups, with no significant difference in incidence between MVM and SVM. Although some studies classified dizziness as a potential serious adverse event, no such events were reported.

## Discussion

This systematic review and meta analysis demonstrates that the MVM is significantly more effective than the SVM in terminating SVT in hemodynamically stable patients presenting to the emergency department. Our results showed that by utilizing the MVM technique, patients with SVT were able to convert to sinus rhythm with a simple, safe, cost-effective, and well-responsive intervention. With the consistency of effect across all included randomized trials and no observed heterogeneity, this suggests that the benefits of the MVM are robust and can be reproducible across varied emergency department settings.

Previous Systematic Review and Meta-Analyses (SRMAs) have yielded similar results on MVM versus SVM on the conversion of SVT to sinus rhythm. One SRMA conducted reported that sinus rhythm was achieved 2.5 times more in the MVM group as compared to the SVM group, thus lowering the need for intravenous medication use [10]. One network meta analysis found that the MVM is 2.7 times more effective than the SVM in converting to sinus rhythm [26]. Another—more recent—network meta analysis conducted in 2025 found that the MVM more than doubled the effectiveness on single attempts to convert SVT to sinus rhythm, as compared to the SVM [27].

Beyond its supremacy in conversion rates to sinus rhythm, the MVM greatly reduces the reliance on pharmacological interventions in the ED for SVT. Intravenous adenosine is the first-line medication for SVT and is effective but carries adverse effects, including chest discomfort, transient arrhythmias, dyspnea, and patient distress [28]. By increasing the likelihood of cardioversion through a non-pharmacological, low-cost, fast, and easily learned maneuver, the MVM offers a safer first-line option that may shorten ED length of stay, decrease the need for intravenous access, and reduce overall resource utilization.

These advantages are particularly meaningful in resource-limited and underserved settings, where access to intravenous antiarrhythmic medications, cardiac telemetry, and specialty consultation may be inconsistent. The MVM requires no equipment beyond a syringe or simple device to generate adequate intrathoracic pressure. The maneuver can be performed by a single clinician at the bedside and does not depend on pharmacy availability of higher-cost medications. Its simplicity and reproducibility make the MVM well suited for rural emergency departments where the burden of arrhythmia care may fall on clinicians with limited resources.

While the MVM remains the most effective and equivalent in terms of safety when performed correctly, there is a higher degree of patient cooperation required for successful performance of this maneuver. As exemplified by Ashraf et al., there have been documented difficulties with patient compliance in language barriers [24]. Similar barriers may exist in ways of age, hearing capabilities, and learning or other developmental disorders.

SVT is the most common arrhythmia in children and commonly presents during adolescence and young adulthood, which is reflected in the age distribution of the populations enrolled in the studies included in this SRMA [29]. Seven of the nine included trials enrolled adult patients beginning at 18 years of age. Of these, two restricted enrollment to patients aged 18–65 years and one to patients aged 18–70 years, while four included adults aged 18 years or older without a specifying upper age limit. One study did not clearly report the age range of its participants, and one pediatric randomized trial enrolled patients aged 7–17 years. This inclusion of pediatric patients provides some presentation of younger patients within the evidence base. Therefore, the relatively young age distribution of the included populations is consistent with the age-related clinical presentation of SVT. However, because only one pediatric trial was included and older adults were less well represented, the generalizability of these finds across the full age spectrum remains limited and warrants future investigations.

Our review offers several methodological advantages over prior analyses. An updated search through March 2026 where several recently published randomized controlled trials were captured, the use of the current Cochrane Risk of Bias 2 tool, and the inclusion of adolescent patients who have been excluded from prior meta-analyses. The pooled analysis yielded an *I*^2^ of 0%, indicating no observed heterogeneity across the nine included trials despite their geographic and demographic diversity.

Several limitations should be acknowledged. First, the review protocol was not prospectively registered. Second, one of the included studies was available only as a conference abstract, restricting the depth of data extraction and risk-of-bias assessment. Although pediatric patients were included, only one pediatric trial met inclusion criteria, limiting the generalizability of these findings to younger populations. Blinding of clinicians and participants to their treatment group was not feasible given the nature of the intervention, although the use of objective ECG confirmation of sinus rhythm mitigates the risk of measurement bias.

## Conclusion

The modified Valsalva maneuver is significantly more effective than the standard Valsalva maneuver for the termination of SVT in hemodynamically stable patients presenting to the ED, without an increase in adverse events. Given its consistent benefit across diverse settings, low cost, and lower risk of adverse effects, the MVM should be considered the routine first-line vagal maneuver in this population. Its simple technique and minimal resource requirements make it especially valuable in the ED, where it can serve underserved populations with limited access to pharmacological alternatives. Future trials should focus on pediatric populations and the direct comparison between the MVM and pharmacological alternatives as first-line therapy in the ED.

## Data Availability

All data generated or analysed during this study are included in this published article.
